# Breakouts—A Radiological Sign of Poor Prognosis in Patients With Brain Metastases

**DOI:** 10.3389/fonc.2022.849880

**Published:** 2022-04-04

**Authors:** Raquel Blazquez, Martin Andreas Proescholdt, Marlene Klauser, Karl-Michael Schebesch, Christian Doenitz, Daniel Heudobler, Lena Stange, Markus J. Riemenschneider, Elisabeth Bumes, Katharina Rosengarth, Andreas Schicho, Nils-Ole Schmidt, Alexander Brawanski, Tobias Pukrop, Christina Wendl

**Affiliations:** ^1^ Department of Internal Medicine III, Hematology and Medical Oncology, University Hospital Regensburg, Regensburg, Germany; ^2^ Department of Neurosurgery, University Hospital Regensburg, Regensburg, Germany; ^3^ Institute of Radiology, University Hospital Regensburg, Regensburg, Germany; ^4^ Department of Neuropathology, University Hospital Regensburg, Regensburg, Germany; ^5^ Department of Neurology and Wilhelm Sander-NeuroOncology Unit, University Hospital Regensburg, Regensburg, Germany

**Keywords:** brain metastasis, infiltration, MRI, rim enhancing, spherical

## Abstract

**Purpose:**

Brain metastases (BM) can present a displacing or infiltrating growth pattern, independent of the primary tumor type. Previous studies have shown that tumor cell infiltration at the macro-metastasis/brain parenchyma interface (MMPI) is correlated with poor outcome. Therefore, a pre-therapeutic, non-invasive detection tool for potential metastatic cell infiltration at the MMPI would be desirable to help identify patients who may benefit from a more aggressive local treatment strategy. The aim of this study was to identify specific magnetic resonance imaging (MRI) patterns at the MMPI in patients with BM and to correlate these patterns with patient outcome.

**Patients and Methods:**

In this retrospective analysis of a prospective BM registry, we categorized preoperative MR images of 261 patients with BM according to a prespecified analysis system, which consisted of four MRI contrast enhancement (CE) patterns: two with apparently regularly shaped borders (termed “rim-enhancing” and “spherical”) and two with irregular delineation (termed “breakout” and “diffuse”). The primary outcome parameter was overall survival (OS). Additionally analyzed prognostic parameters were the Karnofsky Performance Index, tumor size, edema formation, extent of resection, and RPA class.

**Results:**

OS of patients with a breakout pattern was significantly worse than OS of all other groups.

**Conclusion:**

Our data show that BM with a breakout pattern have a highly aggressive clinical course. Patients with such a pattern potentially require a more aggressive local and systemic treatment strategy.

## Introduction

Brain metastases (BM) are the most common form of brain neoplasms in adults and mostly originate from primary tumors of the lung, breast, kidney, or skin (1–3). Although advances in systemic therapy have improved control of BM and subsequently prolonged overall survival (OS), an increased incidence of BM has been reported for several solid tumors (4). Modern imaging techniques, particularly magnetic resonance imaging (MRI), have increased the rate of BM detection in patients with cancer, therefore contributing to the increased incidence of BM (5). MRI is the current gold standard for detecting BM because of its high sensitivity and excellent spatial resolution (6). Routine MRI protocols for BM imaging consist of T2- and T1-weighted sequences with and without the administration of a gadolinium-containing contrast agent. Because of the increased permeability of the tumor vasculature (7), the contrast agent leaks into the interstitial space, causing enhanced T1 signaling, particularly in the peripheral segment of the BM bordering normal brain tissue (8). The resulting enhancement pattern can be spot-like, solid, and homogeneous, or ring-shaped in appearance; the ring shape is caused by central hypoxic necrosis starting at a certain time point and by the volume of the metastases during metastatic progression (9). On T2-weighted images, BM are mostly spherical in shape with well-circumscribed margins and display variable signal intensity due to central hemorrhage, calcification of the BM, cystic portions, or necrosis (10).

Historically, BM were considered to be sharply demarcated lesions without significant infiltration of the host organ (11, 12). In contrast, the histopathological results of more recent studies have shown a significant degree of tumor cell infiltration into the adjacent brain parenchyma (13–15). This observation is supported by significant local recurrence rates after the resection of contrast-enhanced areas in BM in patients who did not receive postsurgical adjuvant radiation, indicating the presence of residual metastatic cells or colonies, even after gross total resection (16, 17). In a recent review, we described several distinct patterns of tumor cell infiltration at the macro-metastasis/brain parenchyma interface (MMPI); such infiltration implicates specific molecular pathways, which generate different types of metastatic brain infiltration (18). A few years ago, a prospective study investigating the impact of tumor cell infiltration in patients with BM not only revealed the presence of tumor cells beyond the glial pseudo-capsule but also showed significantly worse OS of such a growth pattern (2-year OS = 6.6% vs. 43.5%; p = 0.009; HR = 3.4) (19). Consequently, pre-therapeutic, non-invasive, radiological detection of potential metastatic cell infiltration into the brain parenchyma would be extremely desirable to help stratify patients and to identify patients who may benefit from more aggressive local therapy of supramarginal resection (20–22) or intensified radiation treatment (23).

A few years ago. Itakura et al. reported three contrast enhancement (CE) patterns for glioblastoma (GBM) based on different MRI features capturing the shape, edge sharpness, and texture (24). The results of this study indicated a correlation of these imaging phenotypes not only with different molecular patterns but also with the probability of survival. In BM, particularly in the peripheral segment bordering normal brain tissue (8), the different distribution of contrast agents may lead to different CE patterns in MRI. To our best knowledge, no study is yet available trying to cluster BM based on their CE pattern.

Thus, we defined specific CE patterns in MRI of BM in analogy to the work of Itakura et al. on GBM. Moreover, we correlated the patterns with clinical parameters such as the primary tumor, the extent of resection, and OS. According to previous histopathological studies (19) and the work of Itakura et al. (24), we hypothesized that metastases infiltrating the adjacent brain parenchyma may display a more aggressive biology than well-demarcated metastases resulting in poor prognosis.

## Methods and Materials

### Patient Population and Ethical Approval

This prospective BM registry of patients undergoing surgical resection was approved by the ethical review boards of the University of Regensburg (protocol no. 19-1333-104). We included all patients with BM who had undergone surgical resection at the Department of Neurosurgery of the University Medical Center Regensburg between 2005 and 2016 regardless of the primary tumor. In the case of multiple metastases, a maximum of two lesions were resected and only lesions causing clinical symptoms. 82.3% of patients had received adjuvant irradiation and 54.2% systemic therapy. The selection criteria for analyzing the imaging pattern included i) at least one histologically confirmed BM, ii) age older than 18 years, and iii) availability of presurgical MRI containing at least one T2-weighted and one T1-weighted CE sequence. Of the initially 272 patients screened, 11 were excluded from analysis because of large areas of hemorrhagic transformation that did not allow any clear identification of the CE pattern.

### Clinical Data

Presurgical functional impairment of the patients was classified with the Karnofsky Performance Index (KPI). Patients were grouped to recursive partitioning analysis (RPA) classes according to age and presurgical KPI. The extent of resection was classified as complete resection if no residual lesion was detectable in the postoperative MRI performed within 72 h after surgical resection. Time of metastasis was categorized into synchronous and metachronous metastases and the metastasis status in solitary (1 BM without any extracranial metastases), singular (1 BM with extracranial metastases), oligometastatic (2–3 metastatic tumors), and multiple (more than 3 metastatic tumors) (25). Clinical follow-up data were obtained from patient records and by contacting the primary care physicians. OS was calculated from the time point of surgical resection of the BM until the death of the patients.

### Imaging Analysis

MR imaging of the included patients was performed according to a standardized scanning protocol; contrast-agent dosing was applied using a weight-adapted regimen. The different CE patterns of BM on MRI were defined by means of a cohort of 20 randomly selected patients with BM, who were also included in the final evaluation cohort (n = 261). In detail, these 20 BM were visually analyzed with regard to sharpness of the CE demarcation line and signal intensity of CE areas and extent of solid enhancing tumor parts/necrosis. Differences in the characteristics of these imaging markers were used to predefine several distinct CE patterns. An MRI-based assignment of all BM (n = 261) to one of the predefined CE patterns was performed by two blinded readers (CW and MK) using consensus rating. In patients with multiple BM, only the BM with the largest diameter in T1-post contrast was used for analysis.

In addition to the CE pattern, a set of three imaging features was determined for each BM: i) maximum diameter, ii) peritumoral edema, and iii) state of necrosis. The maximum diameter of BM in the CE sequences and the extent of the peritumoral edema in a T2-weighted image were manually measured using presurgical MRI scans in the axial plane. The area of edema around a BM was rated as “large” if its maximum diameter according to the T2-weighted axial images exceeded twice the diameter of the metastasis itself. Otherwise, it was rated as “small.” The existence of central necrosis and hemorrhagic transformation was also assessed combining the image information of T2, T1, and/or T2* images.

### Statistical Analysis

For descriptive statistics, continuous values are reported as mean, median, and range; ordinal and categorical variables are stated in counts and percentages. OS was calculated using the Kaplan–Meier method; univariate analysis of factors associated with survival was performed using log-rank testing. Multivariate analysis of independent prognostic factors was done with cox proportional hazards modeling. Factors which showed statistically significant results in the univariate analysis were included in the multivariate analysis. The presurgical KPI was not included since it is an integral component of the RPA classification and therefore redundant in the multivariate analysis. Validation of proportionality assumption was performed using Schoenfeld residuals (p = 0.658). Results were considered statistically significant with a p-value of <0.05. All analyses were performed using Stata/IC (version 16.1, Stata Corp., College Station, USA).

## Results

### Description of CE Patterns in Human BM

**Figure 1:**
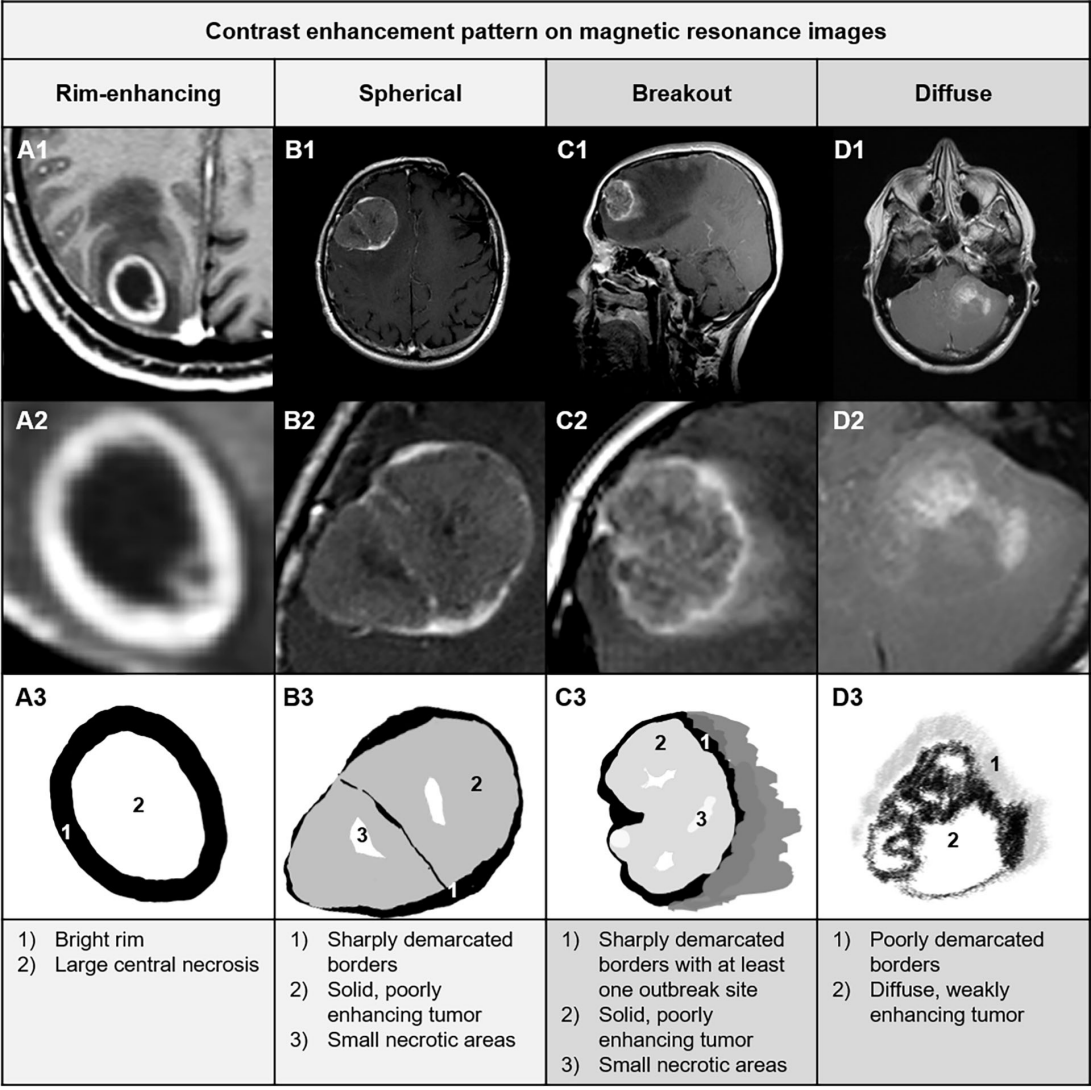
Definition, examples (A1–D1) and description (A2–D2) of the four different MRI subtypes: **(A)** rim-enhancing, **(B)** spherical, **(C)** breakout, and **(D)** diffuse.

Analysis of the MR images of BM yielded four different CE patterns which are described in detail in **Figure 1**. BM with a bright CE ring-like structure (rim), a sharp CE demarcation line, and a large central non-enhancing area (necrosis) were assigned to the subgroup “rim-enhancing CE pattern” (**Figure 1A**). Metastases showing a sharp CE demarcation line, a solid but poor-enhancing tumor within the contrast demarcation line, and relatively small necrosis were classified as spherical (**Figure 1B**). We also identified some metastases with poorly demarcated CE demarcation lines showing a rim-like enhancement which was interrupted at least at one position. This CE pattern was termed breakout (**Figure 1C**). Finally, metastases with blurry borders without any assessable well-demarcated line were allotted to the subgroup “diffuse CE pattern” (**Figure 1D**).

### An MRI Breakout Pattern Is an Independent Factor Associated With Poor OS

**Figure 2:**
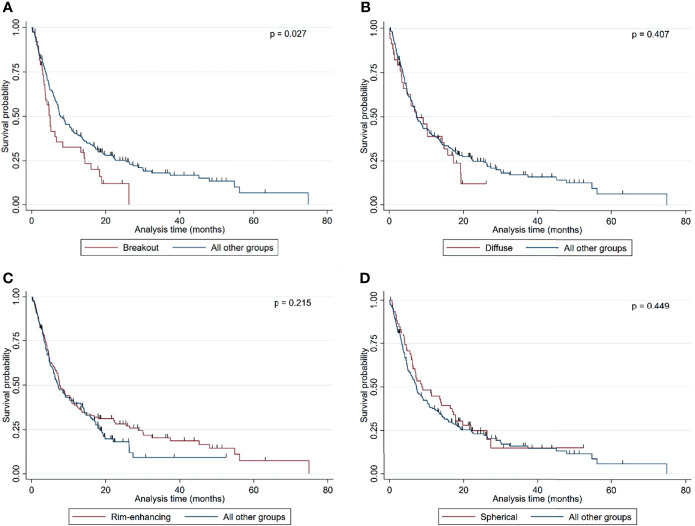
Kaplan–Meier curves showing the overall survival probability stratified by **(A)** breakout vs. all other MRI subgroups, **(B)** diffuse vs. all other MRI subgroups, **(C)** rim-enhancing vs. all other MRI subgroups, and **(D)** spherical vs. all other MRI subgroups. Statistical analysis was performed by calculating log-rank analyses.

Of the 261 metastases screened, 129 had a rim-enhancing CE pattern (49.4%), 59 a spherical pattern (22.6%), 39 a breakout pattern (14.9%), and 34 a diffuse CE pattern (13.0%) (**Table 1**). At the time of analysis, 203 patients (77.8%) had died. The median overall survival time of all patients was 7.23 months from the time of surgical resection (**Table 1**). Interestingly, patients with BM in the breakout group had significantly shorter OS than all other groups (4.71 vs. 7.52 months, respectively; p = 0.027, **Table 1** and **Figure 2**). In an all-pairwise comparison, patients in the breakout group showed a significantly shorter survival compared to the rim-enhancing (p = 0.038) and the spherical group (p = 0.027; **Table 2** and **Supplement Figure 1**). The clinical parameters of the breakout subgroup were similar to those of all other groups except for a significantly larger tumor size (p = 0.034) and a lower number of metastases (p = 0.021) (**Table 3**). Furthermore, the breakout pattern was not associated with the primary tumor (p = 0.584; **Table 4**).

**Table 1:** Overall survival stratified according to the contrast enhancement pattern on magnetic resonance images.

Parameter	Frequency [number (%)]	2-year survival rate [%]	Overall survival [median; months]	95% CI (of the median OS)		p-value (selected group vs. all other groups)
**CE pattern**						
**All**	261	13.8	7.23	0.897	37.413	0.215
**Rim-enhancing**	129 (49.4)	27 (20.9)	7.56	1.084	48.558	0.449
**Spherical**	59 (22.6)	6 (10.7)	8.77	0.821	30.608	0.027
**Breakout**	39 (14.9)	2 (5.1)	4.71	0.897	16.460	0.407
**Diffuse**	34 (13.0)	1 (2.9)	6.32	0.891	19.528	

**Table 2:** All pairwise comparison of overall survival according to the contrast enhancement pattern on magnetic resonance images.

Contrast enhancement pattern	Hazard ratio	95% CI		p-value
Breakout vs. rim-enhancing	1.543	1.024	2.236	0.038
Rim-enhancing vs. diffuse	1.118	0.895	1.396	0.323
Rim-enhancing vs. spherical	0.995	0.883	1.120	0.936
Breakout vs. diffuse	0.821	0.481	1.399	0.469
Breakout vs. spherical	0.767	0.606	0.970	0.027
Diffuse vs. spherical	0.736	0.446	1.213	0.230

**Table 3:** Clinical characteristics of the entire patient cohort stratified according to the breakout pattern and all other groups.

Parameter	Entire population [number (%)]	Contrast enhancement pattern [number (%)]		p-value
		Breakout	All other groups	
**N =**	261	39 (14.9)	222 (85.1)	
**Age** **(years; median)**	61.5	58.8	61.6	0.519
**Sex (f/m)**	127 (48.6)/134 (51.4)	19 (48.7)/20 (51.3)	108 (48.7)/114 (51.3)	0.994
**Tumor size** **(mm; median)**	29	32	28	0.034
**Edema**	157 (60.2)	26 (66.7)	131 (59.0)	0.364
**Hemorrhage**	19 (7.3)	3 (7.7)	16 (7.2)	0.941
**Time of metastasis**				
**Synchronous**	92 (35.2)	11 (28.2)	81 (36.5)	0.318
**Metachronous**	169 (64.8)	28 (71.8)	141 (63.5)	
**Metastasis status*^a^***				
**Solitary**	64 (24.5)	9 (23.1)	55 (24.7)	0.208
**Singular**	84 (32.2)	18 (46.2)	66 (29.7)	
**Oligo**	71 (27.2)	8 (20.5)	63 (28.4)	
**Multiple**	42 (16.1)	4 (10.2)	38 (17.2)	
**Number of metastases** **(median; range)**	1.0 (1–6)	1.0 (1–4)	1.0 (1–6)	0.021
**Presurgical KPI (median)**	90	90	90	0.655
**Extent of resection**				0.487
**Complete**	211 (80.8)	32 (82.1)	179 (80.6)	
**Incomplete**	50 (19.2)	7 (17.9)	43 (19.4)	
**RPA class**				0.218
**I**	40 (15.3)	9 (23.1)	31 (14.0)	
**II**	194 (74.3)	28 (71.8)	166 (74.7)	
**III**	27 (10.4)	2 (5.8)	25 (11.3)	

^a^Solitary: 1 BM without extracranial metastases; singular: 1 BM with extracranial metastases; oligometastatic: 2–3 BM; multiple: >3 BM.

**Table 4:** Distribution of primary tumors within the entire cohort stratified into breakout pattern and all other groups.

Primary tumor	Entire population[number (%)]	Contrast enhancement pattern[number (%)]		p-value
		Breakout	All other groups	
**N =**	261	39	222	
Lung cancer	94 (36)	17 (43.6)	77 (34.6)	0.584
Breast cancer	40 (15.3)	5 (12.8)	35 (15.8)	
Melanoma	40 (15.3)	5 (12.8)	35 (15.8)	
Colon cancer	20 (7.7)	3 (7.7)	17 (7.6)	
Renal cell cancer	14 (5.4)	1 (2.5)	13 (5.9)	
CUP	13 (5.0)	0 (0.0)	13 (5.9)	
Prostate cancer	6 (2.3)	2 (5.1)	4 (1.8)	
Cervical cancer	5 (1.9)	0 (0.0)	5 (2.3)	
Urothelial cancer	5 (1.9)	1 (2.5)	4 (1.8)	
Gastric cancer	3 (1.2)	0 (0.0)	3 (1.3)	
Others*^a^*	21 (8.1)	5 (12.8)	16 (7.2)	

CUP, cancer of unknown primary.

^a^“Others” refers to rare tumor entities such as sarcoma, hepatocellular carcinoma, and ovarian and testicular cancer.

In contrast to the breakout pattern, complete resection (p = 0.023), good presurgical KPI (p = 0.011), low RPA class (p = 0.003), and solitary metastatic status (p = 0.001) were associated with better OS in the univariate analysis (**Table 5** and **Supplement Figure 2**). This finding is in accordance with a series of studies of patients with BM (26–29), indicating that our cohort is representative and not subject to significant selection bias. In contrast, OS was not significantly correlated with age (p = 0.594), sex (p = 0.694), primary tumor type (p = 0.891), time of metastasis (synchronous vs. metachronous; p = 0.734), number of metastases (p = 0.150), size of the resected metastatic tumor (p = 0.072), or presence of necrosis (p = 0.299), hemorrhage (p = 0.505), or large edema (p = 0.307) (**Table 5**).

**Table 5:** Univariate analysis of factors associated with overall survival in the entire cohort.

Parameter	Hazard ratio	95% CI		p-value
MRI CE pattern (breakout vs all other groups)	1.744	1.190	2.556	0.027
Extent of resection (complete vs. incomplete)	0.679	0.485	0.950	0.023
Presurgical KPI (≥70 vs. <70)	0.622	0.406	0.953	0.011
RPA class (class I vs. all others)	0.655	0.493	0.868	0.003
Metastasis status (solitary vs. all others)	0.755	0.642	0.888	0.001
Age	1.006	0.993	1.019	0.594
Sex	1.006	0.994	1.020	0.694
Primary tumor	0.979	0.929	1.031	0.891
Time of metastasis class (synchronous vs. metachronous)	0.931	0.699	1.238	0.734
Number of metastases	1.149	1.037	1.274	0.150
Size of resected tumor	1.013	1.002	1.024	0.072
Necrosis	0.795	0.579	1.092	0.299
Hemorrhage	0.838	0.495	1.471	0.505
Large edema	0.888	0.672	1.174	0.307

Finally, multivariate cox hazard regression analysis showed the breakout pattern (p = 0.003) and other well-established prognostic markers such as the solitary metastatic status (p = 0.005), good RPA class (p = 0.022), and complete resection (p = 0.030) to be independent factors associated with poor OS (**Table 6**).

**Table 6:** Multivariate analysis of independent factors associated with overall survival.

Parameter	Hazard ratio	95% CI		p-value
CE pattern (breakout vs. all other groups)	1.836	1.224	2.752	0.003
Extent of resection (incomplete vs. complete)	0.685	0.487	0.964	0.030
Metastasis status (solitary vs. all others)	0.774	0.646	0.926	0.005
RPA class (class I vs. all others)	0.664	0.468	0.943	0.022

## Discussion

The aim of the present study was to define radiological MRI patterns of BM at the MMPI and to correlate these patterns with OS. Previous MRI studies of BM analyzed general morphological parameters, such as the shape of the lesion, edema formation, or the area of general contrast enhancement. Thereby, the MMPI was overlooked, and these parameters were not correlated with OS (30). Based on the fact that BM can present as either infiltrating or displacing (15, 18, 19) and in accordance with Itakura’s radiological segregation of GBM (24), we hypothesized that there must be different recognizable BM subtypes in CE patterns on MRI scans.

Our previous histological analysis of BM at the MMPI showed that displacing metastases may or may not be surrounded by a highly vascularized glial pseudo capsule (18, 19). Itakura et al. also described two GBM subtypes with regular edges: with or without a hypointense center (rim enhancing and spherical) (24). Thus, we assumed that these two different patterns should also be detectable in BM by means of MRI scans. In fact, we identified two CE patterns with regular borders, which were found in the majority of our study patients: one group with sharply demarcated edges with a bright rim and a necrotic and therefore hypointense core (rim-enhancing) and another group without a hyperintense rim around the solid metastasis (spherical). These two patterns resembled the patterns described by Itakura et al. for GBM.

Furthermore, our histological analysis of BM at the MMPI also revealed different MMPI patterns for infiltrative metastases. For example, we observed that epithelial infiltrative BM break through the glial-pseudo capsule with multiple strands and cohorts of metastatic cells, often only at one site in the entire circumvention. This MMPI pattern completely differs to BM with diffuse infiltrative and angiotropic MMPIs, where single tumor cells or small groups of cells can often be seen at a distance from the metastatic core (18, 19). Therefore, we assumed that metastases with diffuse infiltrative or angiotropic MMPIs should have a different radiological CE pattern at the MMPI than BM with an epithelial infiltrative MMPI. The study by Itakura et al. identified only one MRI pattern with irregular borders, which they termed “pre-multi-focal” (24). In our study, we broadened the observations made by Itakura et al. and identified two instead of one irregularly shaped CE pattern on MRI, as expected from our histopathological studies. First, we describe a breakout pattern with poorly demarcated CE instead of interrupted sharp rim-like enhancement at least at one position. In addition, we also describe a diffuse pattern without any assessable demarcation line.

One of our previous histopathological studies showed that infiltrative BM have worse OS than displacing BM (19). Itakura et al. also correlated the pre-multi-focal pattern with worse OS (24). Consequently, we hypothesized that BM showing irregular borders on MRI scans (breakout and/or diffuse pattern) may display a more aggressive biology than sharply demarcated BM (rim-enhancing and/or spherical pattern) and result in worse patient outcome. Indeed, our results showed the breakout pattern to be an independent prognostic factor for poor survival. The validity of these data is reinforced by the observations that other well-known prognostic markers such as solitary metastatic status and complete resection (26, 28, 29, 31) have also been identified as independent prognostic factors associated with better OS in this cohort. Furthermore, the breakout pattern in BM was independent of the primary tumor. Some primary tumors are well known for their association with poor outcome. For example, a retrospective study including 740 patients showed the highest survival rate (23.9%) 2 years after the diagnosis of BM for patients with ovarian carcinoma and the lowest survival rate (1.7%) for patients with small cell lung cancer (32). However, the breakout pattern identified in our BM cohort was not associated with any primary tumor, highlighting the irrefutable value of such a biomarker for patient stratification and clinical decision-making. The patients displaying a breakout pattern did not significantly differ in most clinical parameters from all other groups except for larger tumor size and a lower number of lesions.

The impact of tumor size in BM remains unclear. One study in patients with breast cancer and BM showed worse outcome of patients with a tumor size larger than 5 cm (33). In contrast, two studies did not yield any correlation between BM size and OS (34, 35). Although we did detect an association between tumor size and OS, this effect did not reach statistical significance, neither in the univariate nor in the multivariate analysis. Regarding the number of lesions, the majority of reports yielded poorer OS with increasing numbers of BM (31, 36–39), which is counterintuitive with regard to patients with a breakout pattern who have significantly shorter OS despite a lower number of lesions. Conceivably, the impact of the breakout pattern on outcome is independent from the number of lesions, but this aspect needs to be validated in a prospective manner.

The second subgroup with irregular edges (diffuse MRI pattern) showed no significant difference in OS to the other groups. One reason for this lack of statistical significance despite a descriptively shorter survival in this group might be the relatively low patient number, potentially leading to insufficient statistical power. Alternatively, it is possible to speculate that some of the diffuse infiltrative BM do not potentially get their nutrients by neovascularization but by co-opting existing blood vessels without inducing a leakage (18). This process results in a potential lack of CE in these areas, which may lead to the incorrect categorization of BM with a diffuse MRI pattern. In this context, Spanberger et al. reported that metastases with small brain edema are significantly correlated with a metastatic brain-infiltrative growth pattern, lower HIF1α expression, and less angiogenetic activity in contrast to metastases with large peritumoral edema. Spanberger et al. hypothesized that vascular co-option and low microvascular density, as seen in the infiltration zone, are potential reasons for small peritumoral edema of infiltrating metastases (40). Thus, the correlation of histological diffuse infiltration on the single-cell level with the pre-interventional MRI seems to be the biggest challenge.

In this study, we identified for the first time four BM subtypes that were solely differentiated by qualitative MRI features and showed the prognostic relevance of the breakout pattern. The correlation of the breakout pattern with poor OS are in alignment with previous observations (18, 19, 24). The easily identifiable focally blurred borders as the hallmark of the breakout pattern are thereby suggestive of compact epithelial infiltrative tumor growth that is within the range of the MRI resolution.

## Conclusion

Our data indicate that the MRI breakout pattern is an imaging biomarker for particularly poor outcome in patients with BM. Prospective trials are underway to analyze the histological and biological MMPI characteristics of patients with BM with an MRI breakout pattern. In addition, we propose that such patients may require more aggressive local and systemic treatment.

## Data Availability Statement

The original contributions presented in the study are included in the article/**Supplementary Material**. Further inquiries can be directed to the corresponding authors.

## Ethics Statement

The studies involving human participants were reviewed and approved by the ethical review board of the Regensburg University Hospital (protocol no. 19-1333-104). Written informed consent for participation was not required for this study in accordance with the national legislation and the institutional requirements.

## Author Contributions

RB, MP, TP, CW: conception and design. RB, MP, MK, K-MS, CD, DH, LS, EB, KR, TP, CW: analysis and interpretation of data. RB, MP, MK, K-MS, CD, DH, LS, MR, EB, KR, AS, N-OS, AB, TP, CW: manuscript draft and/or revision. All authors contributed to the article and approved the submitted version.

## Funding

This work was supported by the German Research Foundation (grants DFG-PU355/4-1 and FOR2127-PU355/5-1).

## Conflict of Interest

The authors declare that the research was conducted in the absence of any commercial or financial relationships that could be construed as a potential conflict of interest.

## Publisher’s Note

All claims expressed in this article are solely those of the authors and do not necessarily represent those of their affiliated organizations, or those of the publisher, the editors and the reviewers. Any product that may be evaluated in this article, or claim that may be made by its manufacturer, is not guaranteed or endorsed by the publisher.

## Supplementary Material

Supplementary Figure 1 Kaplan-Meier curves showing the overall survival probability in an all – pairwise comparison between the four predefined CE pattern. **(A)** Rim-enhancing vs. breakout; **(B)** Rim-enhancing vs. diffuse; **(C)** Rim-enhancing vs. spherical; **(D)** Breakout vs. diffuse; **(E)** Breakout vs. spherical and **(F)** Diffuse vs. spherical.

Supplementary Figure 2 Kaplan-Meier curves showing the overall survival probability stratified by **(A)** extent of resection consisting of complete (blue curve) and incomplete (red curve) resection, **(B)** overall survival times in months correlated to the presurgical KPI classes, **(C)** RPA class, and **(D)** metastasis status consisting of solitary (one BM, no extracranial metastases), singular (one BM combined with extracranial metastases), oligo (2-3 BM) and multiple metastases (> 3 BM). Statistical analysis was performed by calculating log rank analyses.

## Abbreviations

BM, brain metastasis; CE, contrast enhancement; CI, confidence interval; f, female; HR, hazard ratio; KPI, Karnofsky Performance Index; m, male; MRI, magnetic resonance imaging; MMPI, macro-metastasis/brain parenchyma interface; OMD, oligo-metastatic disease; OS, overall survival; RPA, recursive partitioning analysis; vs., versus.
