# Supplementary figures and images for: Breakouts—A Radiological Sign of Poor Prognosis in Patients With Brain Metastases
Source: Front Oncol. 2022 Apr 4;12:849880. doi: 10.3389/fonc.2022.849880 (PMC9015662; doi:10.3389/fonc.2022.849880)

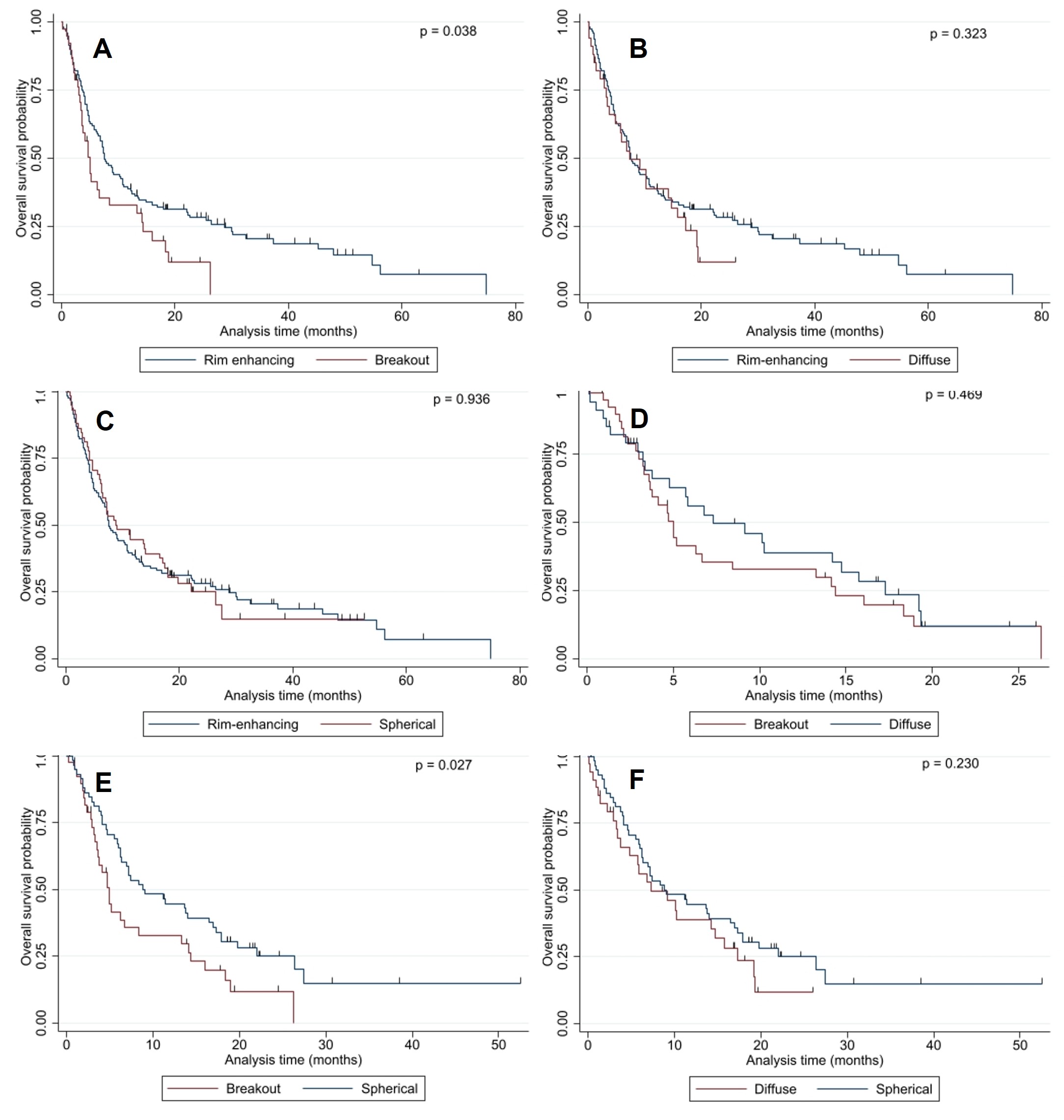

Supplement: Supplementary Figure 1 — Kaplan-Meier curves showing the overall survival probability in an all – pairwise comparison between the four predefined CE pattern. (A) Rim-enhancing vs. breakout; (B) Rim-enhancing vs. diffuse; (C) Rim-enhancing vs. spherical; (D) Breakout vs. diffuse; (E) Breakout vs. spherical and (F) Diffuse vs. spherical. [file Image_1.jpeg]

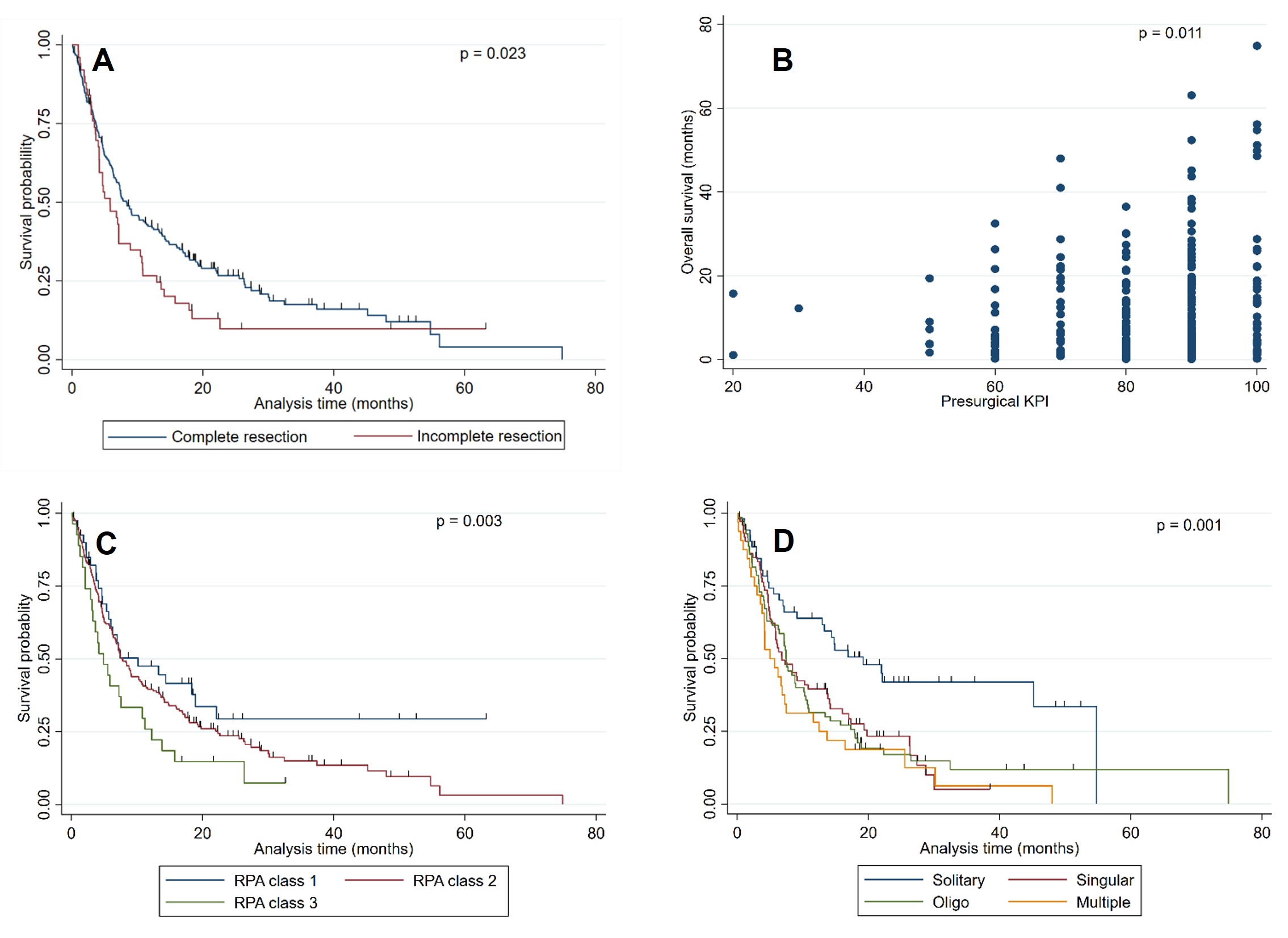

Supplement: Supplementary Figure 2 — Kaplan-Meier curves showing the overall survival probability stratified by (A) extent of resection consisting of complete (blue curve) and incomplete (red curve) resection, (B) overall survival times in months correlated to the presurgical KPI classes, (C) RPA class, and (D) metastasis status consisting of solitary (one BM, no extracranial metastases), singular (one BM combined with extracranial metastases), oligo (2-3 BM) and multiple metastases (> 3 BM). Statistical analysis was performed by calculating log rank analyses. [file Image_2.jpeg]
